# Uncovering the molecular mechanisms of Qingdu Zengye Decoction in the treatment of nasopharyngeal carcinoma: an integrative investigation

**DOI:** 10.3389/fphar.2025.1648294

**Published:** 2025-07-17

**Authors:** Qi Quan, Zeyu Liu, Ran Ding, Yongmiao Lin, Sihe Zhang, Wei Luo, Mengjie Lei, Teng Fan, Xin Su, Yuanyuan Huang, Roujun Peng, Bei Zhang

**Affiliations:** ^1^ State Key Laboratory of Oncology in South China, Guangdong Key Laboratory of Nasopharyngeal Carcinoma Diagnosis and Therapy, Guangdong Provincial Clinical Research Center for Cancer, Sun Yat-sen University Cancer Center, Guangzhou, China; ^2^ Integrated Traditional Chinese and Western Medicine Research Center, Guangzhou, China; ^3^ Department of Cell Biology, School of Medicine, Nankai University, Tianjin, China; ^4^ Institute of Clinical Medicine, The First Affiliated Hospital of University of South China, Hengyang, China

**Keywords:** nasopharyngeal carcinoma, Qingdu Zengye Decoction, molecular networks, Chinese herb medicine, therapeutic mechanism

## Abstract

**Background:**

Nasopharyngeal carcinoma (NPC) remains a therapeutic challenge due to its aggressive nature and limited treatment efficacy. Traditional Chinese Medicine, particularly Qingdu Zengye Decoction (QZD), has shown clinical potential, but its mechanistic basis in NPC treatment requires elucidation.

**Purpose:**

This study aims to elucidate the mechanisms of action of QZD in the treatment of NPC, focusing on its multi-target regulatory effects on cell apoptosis, oncogenic signaling pathways, and tumor immune microenvironment.

**Methods:**

An integrative approach combining computational pharmacology, functional experiments, and single-cell transcriptomic profiling was employed to dissect QZD’s anti-NPC mechanisms. Network pharmacology and protein-protein interaction (PPI) analysis was used to identify potential QZD targets. Functional assays (cell proliferation, apoptosis, colony formation) and Western blotting were used to validate key pathways. Molecular docking was applied to assessed ligand-target binding affinities. Single-cell RNA sequencing (scRNA-seq) was used to analyzed spatial expression patterns in NPC tumor samples.

**Results:**

QZD suppressed tumor progression by inducing apoptosis through modulating Bax in a dose-dependent manner and inhibiting the PI3K-Akt signaling pathway. Network pharmacology analysis identified AKT1, MTOR, HIF1A, SRC, and ESR1 as core regulatory genes. scRNA-seq revealed compartment-specific target localization: AKT1/ESR1 in tumor cells, SRC/IL6 in myeloid cells, and MTOR/HIF1A across stromal compartments. Molecular docking confirmed strong interactions between QZD compounds (e.g., quercetin, luteolin) and these targets. Upregulation of IL6 was observed and its dual immune-modulatory effects involving tumor suppression and microenvironment reprogramming was suggested.

**Conclusion:**

QZD exerts anti-tumor effects in NPC through apoptosis induction, PI3K-Akt pathway suppression, and multi-compartmental tumor microenvironment modulation. Its ability to concurrently target oncogenic signaling and immune regulation positions QZD as a promising therapeutic strategy for advanced NPC.

## Introduction

Nasopharyngeal carcinoma (NPC) is a malignant tumor originating in the nasopharynx, with a notably high prevalence in Southern China (Chen et al., 2019; Tang et al., 2022). The development of NPC is strongly linked to Epstein-Barr virus (EBV) infection. For locally advanced NPC, the standard treatment involves induction chemotherapy combined with concurrent chemoradiotherapy (CCRT), which results in a five-year survival rate exceeding 80% (Liu et al., 2024a). However, despite these advancements, the rate of relapse and metastasis remains high in patients with high risk of recurrence, with approximately 30% of patients experiencing local recurrence or distant metastasis. This compromising long-term disease control, highlighting the continued challenges in achieving long-term survival (Zhang et al., 2019; Liu et al., 2023). Consequently, improving treatment efficacy and reducing recurrence and metastasis in high-risk patients remains a critical area of NPC research.

In recent years, chemotherapy has been used for high-risk, locally advanced NPC patients (Lee A. W. et al., 2019; Liu S. L. et al., 2024; Tan et al., 2018). However, regarding adjuvant therapy after chemoradiotherapy, previous studies indicate that PF (cisplatin + fluorouracil) or GP (gemcitabine + cisplatin) adjuvant chemotherapy does not significantly improve survival, because patients often exhibit poor tolerance to these chemotherapy regimens (Zhang et al., 2016; Li W. Z. et al., 2022; Mai et al., 2023; Hong et al., 2021). A prospective study from Ma Jun’s team demonstrated that metronomic capecitabine therapy after chemoradiotherapy significantly improved survival for high-risk recurrence patients (Chen YP. et al., 2021). Recent research has also focused on the clinical value of anti-PD-1 antibody interventions. The CONTINUUM and DIPPER studies suggest that concurrent or post-chemoradiotherapy anti-PD-1 antibody therapy can reduce the risk of recurrence and death of high-risk patients (Liu et al., 2024a; Liang YL. et al., 2025). However, the use of anti-PD-1 antibodies combined with chemotherapy may result in immune-related adverse reactions and hand-foot syndrome, with some patients needing to discontinue treatment due to intolerability.

Traditional Chinese Medicine (TCM), a distinctive medical system, has gained recognition and widespread use in cancer treatment and has attracted increasing international attention (Yanwei et al., 2022; Chen and Liu, 2024; Xi et al., 2025). Surveys suggest that approximately 80% of cancer patients in China receive TCM therapy, which not only improves treatment outcomes and reduces adverse reactions but also lowers treatment costs and enhances patients’ quality of life (Li et al., 2025). From the TCM perspective, NPC patients post-chemoradiotherapy typically present with Qi and Yin deficiency, along with *phlegm heat and stasis toxins*. Qingdu Zengye Decoction (QZD), a classic TCM formula developed by our team at Sun Yat-sen University Cancer Center, is composed of TaiZishen (Pseudostellaria heterophylla), Xuanshen (Scrophularia ningpoensis), ShengDihuang (Rehmannia glutinosa), Maidong (Ophiopogon japonicus), TianNanxing (Rhizoma Arisaematis), ShiShangbai (Selaginella doederleinii Hieron), Chonglou (Rhizoma Paridis), and Wugong (Centipede) (Liang H. et al., 2025; Liu et al., 2011; Sui et al., 2016; Dong et al., 2021). Based on the core principles of supplementing Qi and nourishing Yin, this formula is designed to resolve phlegm, dissipate nodules, remove blood stasis, unblock collaterals, clear heat, and detoxify. In our clinical observations, post-chemoradiotherapy administration of QZD has shown promise in shrinking residual tumor lesions and extending survival time. However, the molecular mechanisms by which QZD exerts its effects in NPC remain unclear. In this study, we aim to uncover the molecular mechanisms underlying the multi-gene regulatory effects of QZD in NPC.

Due to the complex compositions of TCM formulas, traditional single-research methods are often insufficient to fully elucidate their mechanisms of action. Multi-omics integrated analysis has emerged as a modern and effective approach to unraveling the mechanisms of action of traditional Chinese medicine. In this study, by analyzing a clinical cohort of recurrent and non-recurrent NPC patients, we identified a set of genes whose expression correlates with the recurrence of NPC. Using multi-omics approaches, including transcriptomic analysis, methylation sequencing, and network pharmacology, we explored the molecular mechanisms underlying QZD’s effects in treating NPC. These findings contribute to a better understanding of the molecular networks involved in TCM therapy and underscore the potential for TCM to serve as an adjunctive treatment in NPC management.

## Methods

### Study subjects and biospecimens

Tissue samples were obtained from the Sun Yat-sen University Cancer Center (SYSUCC; Guangzhou, China), including 9 paraffin-embedded specimens from patients who experienced tumor recurrence within 5 years after radiotherapy, as well as 9 samples from patients without recurrence (Human ethics approval code: No. B2022-313-01 Date:2022-05-18). The demographic characteristics and clinical data of the participants were retrieved from medical records (Supplementary Table S1). The study protocol was approved by the Institutional Ethical Review Board of Sun Yat-sen University Cancer Center (No. B2022-313-01), and all patients provided written informed consent for the use of their tissue samples upon admission. All procedures, including the use of human tissue specimens and analysis of clinical data, were conducted in strict accordance with the guidelines of the Declaration of Helsinki.

### Reagents and instrumentation for UHPLC-OE-MS-based untargeted metabolomics

The untargeted metabolomics analysis was performed using ultra-high-performance liquid chromatography coupled with high-resolution mass spectrometry (UHPLC-OE-MS). The following high-purity reagents were used: methanol (LC-MS grade, CNW Technologies, CAS: 67-56-1), acetonitrile (LC-MS grade, CNW Technologies, CAS: 75-05-8), hydrochloric acid (analytical grade, Titan, CAS: 7647-01-0), sodium chloride (analytical grade, Sangon Biotech, CAS: 7647-14-5), ultrapure water (Watsons), acetic acid (LC-MS grade, Sigma-Aldrich, CAS: 64-19-7), and 2-propanol (LC-MS grade, CNW Technologies, CAS: 67-63-0).

Sample preparation and metabolite extraction were conducted using a JXFSTPRP-24 homogenizer (Shanghai Jingxin Technology Co., Ltd.) and a PS-60AL ultrasonic processor (Shenzhen Leidebang Electronics Co., Ltd.). Centrifugation was performed using a Heraeus Fresco17 refrigerated centrifuge (Thermo Fisher Scientific). The extracts were concentrated using an LGJ-10C freeze dryer (Sihuan Furuike Instrument Technology Development Co., Ltd.).

Chromatographic separation was carried out on a Vanquish UHPLC system (Thermo Fisher Scientific), and high-resolution mass spectrometric detection was performed using an Orbitrap Exploris 120 mass spectrometer (Thermo Fisher Scientific). Sample weights were accurately measured using a BSA124S-CW analytical balance (Sartorius). All procedures were conducted following standard protocols to ensure reproducibility and analytical accuracy.

### Components extraction from QZD solutions

The QZD solution was centrifuged at 12,000 rpm (RCF = 13,800 (× g), R = 8.6 cm) for 15 min at 4°C. 300 μL of the supernatant liquids was taken, mixed with 1,000 μL of extraction solution (MeOH:ACN:H2O, 2:2:1 (v/v/v)) which contain deuterated internal standards. The mixed solutions were vortexed for 30 s, sonicated for 5 min in 4°C water bath and incubated for 1 h at −20°C to precipitate proteins. Then the samples were centrifuged at 12,000 rpm (RCF = 13,800 (× g), R = 8.6 cm) for 15 min at 4°C. The supernatants were transferred to fresh glass vials for analysis. The quality control sample was prepared by mixing an equal aliquot of the supernatant of samples.

### LC-MS/MS analysis

LC-MS/MS analyses were performed using an UHPLC system (Vanquish, Thermo Fisher Scientific) with a Phenomenex Kinetex C18 (2.1 mm × 100 mm, 2.6 μm) coupled to Orbitrap Exploris 120 mass spectrometer (Orbitrap MS, Thermo). The mobile phase A:0.01% acetic acid in water; mobile phase B:IPA:ACN (1:1,v/v). The auto-sampler temperature was 4°C, and the injection volume was 2 μL. The Orbitrap Exploris 120 mass spectrometer was used for its ability to acquire MS/MS spectra on information-dependent acquisition mode in the control of the acquisition software (Xcalibur, Thermo). In this mode, the acquisition software continuously evaluates the full scan MS spectrum. The ESI source conditions were set as following: sheath gas flow rate 50 Arb, Aux gas flow rate 15 Arb, capillary temperature 320°C, full MS resolution 60,000, MS/MS resolution 15,000, collision energy: SNCE 20/30/40, spray voltage 3.8 kV (positive) or −3.4 kV (negative).

### Data preprocessing and annotation

The raw data were converted to the mzXML format using ProteoWizard and processed with an in-house program. which was developed using R and based on XCMS, for peak detection, extraction, alignment, and integration. The R package and the BiotreeDB (V3.0) were applied in metabolite identification.

### Screening of disease-related genes for NPC

The disease-related genes for NPC were retrieved from the GeneCards database using “nasopharyngeal carcinoma” as the keyword. The intersection between the drug target genes and NPC-related genes was identified using Venny 2.1, resulting in a set of potential therapeutic targets for NPC.

### Construction of the drug-component-target-disease network

The relationships among the herbal medicine, active components, target genes, and NPC were integrated and visualized using Cytoscape 3.9.1. The NetworkAnalyzer tool was employed to calculate the topological properties of the network, and the final “Drug-Component-Target-Disease” network was generated after optimization.

### Construction of protein-protein interaction (PPI) network and identification of core target genes

The potential therapeutic targets for NPC were imported into the STRING database (version 11.5) with the following parameters: species set to “*Homo sapiens*,” minimum interaction score >0.4, and removal of disconnected nodes. The resulting PPI network was exported in TSV format and imported into Cytoscape 3.9.1 for further analysis. The topological properties of the network were calculated using the NetworkAnalyzer tool. Six topological parameters, including Betweenness Centrality (BC), Closeness Centrality (CC), Degree Centrality (DC), Eigenvector Centrality (EC), Network Centrality (NC), and Local Average Connectivity (LAC), were computed using the cytoNCA plugin. Nodes with higher parameter values were considered more critical in the network, and the core target genes were identified accordingly.

### Genomic DNA extraction and methylation analysis

Genomic DNA was extracted from tissue specimens utilizing the Allprep DNA/RNA Kit (Qiagen). Genome-wide methylation profiling was conducted using the Infinium HumanMethylation850 BeadChips (850K array, Illumina). Data preprocessing, normalization, and β-value computation were executed with the R package minfi (v1.26.2). Gene annotations were segmented into six regions: TSS1500, TSS200, 5′UTR, 1st Exon, gene body, and 3′UTR. CpG island annotations were divided into five regions: N shelf, N shore, CpG Island, S shore, and S shelf.

Quality control measures included: (i) removal of probes with detection P-values ≥0.01 in over 5% of samples; (ii) exclusion of probes on the X or Y chromosomes; (iii) elimination of probes overlapping SNPs; and (iv) discarding probes mapping to multiple genomic loci. Post-quality control, 866,092 probes were retained for subsequent analysis.

CpG probe annotations were sourced from the ENCODE Project database. Each probe was annotated with corresponding gene, genomic region, CpG island-associated regions, and functional regions.

### Differentially methylated CpG sites identification

Differentially methylated CpG sites and regions were identified using the IMA package in R. The analysis was conducted based on both gene annotation categories (TSS1500, TSS200, 5′UTR, 1st Exon, gene body, and 3′UTR) and CpG island annotation categories (N shelf, N shore, CpG Island, S shore, and S shelf). For each region, the signal values of all probes were averaged, and differential methylation regions (DMRs) were identified using the pooled t-test method. DMRs were selected based on the following criteria: P < 0.05 and |Beta.Difference| > 0.14.

The Beta value (β), which ranges between 0 and 1, represents the methylation level at a given CpG site. Values closer to 1 indicate higher methylation, while values closer to 0 indicate lower methylation. The Beta value was calculated using the following formula:

$${\text{beta}}_{i}=\frac{\max\left(y_{\left(i,\text{methy}\right)},0\right)}{\max\left(y_{\left(i,\text{methy}\right)},0\right)+\max\left(y_{\left(i,\text{unmethy}\right)},0\right)+100}$$

The Beta value density curves of the samples were generated to visualize the overall methylation distribution of all CpG sites within each sample, enabling the identification of potential differences in global methylation patterns across high-risk locally advanced NPC sample groups.

### GO and KEGG enrichment analysis

The potential therapeutic targets for NPC were subjected to Gene Ontology (GO) functional enrichment analysis and Kyoto Encyclopedia of Genes and Genomes (KEGG) pathway enrichment analysis using the DAVID database. For GO analysis, the top 10 biological processes with the lowest P-values were selected and visualized using bar plots. For KEGG analysis, the top 20 significantly enriched pathways were identified and visualized using bubble plots.

### Single-cell processing of fresh biopsies

A total of 15 primary NPC tumor samples were collected for single-cell RNA sequencing (scRNA-seq). Fresh biopsy samples designated for scRNA-seq were thoroughly washed with phosphate-buffered saline (PBS; Thermo Fisher Scientific) and minced into fragments of less than 1 mm^3^. The tissue fragments were subsequently transferred into 5 mL of Dulbecco’s Modified Eagle Medium (DMEM) supplemented with collagenase IV (1 μg/mL; Thermo Fisher Scientific) and incubated at 37°C for over 40 min, with manual agitation every 10 min to facilitate enzymatic digestion. Following digestion, the cell suspension was filtered using 5-mL round-bottom polystyrene test tubes equipped with cell strainer snap caps (BD-Falcon). The filtrate was then centrifuged at 800 rpm for 5 min, after which the resulting cell pellet was collected and resuspended in a cell preservation solution (CELLBANKER 2). Cell viability and concentration were subsequently determined via Trypan blue staining using a hemocytometer. The entire single-cell processing workflow was completed within 90 min to ensure optimal cell integrity.

### 10X genomics chromium gene expression library preparation, sequencing, and data processing

The prepared single-cell suspensions were loaded onto the 10X Chromium system for single-cell RNA sequencing, utilizing either the Chromium Single Cell 3′ Library & Gel Bead Kit v2 or v3 (10X Genomics) or the 5′ Library & Gel Bead Kit (10X Genomics). Each well was designed to capture between 8,000 and 14,000 cells. Library preparation was conducted following the manufacturer’s protocols, ensuring high-quality gene expression profiling. Sequencing was performed on an Illumina HiSeq X Ten platform using a 150-bp paired-end read configuration.

For quality control, unique molecular identifiers (UMIs) and mitochondrial genes were quantified using Seurat (version 4.3.0). Cells with more than 100 UMIs and less than 15% of UMIs derived from mitochondrial genes were selected for analysis. In this study, the top 20 principal components and the top 2000 highly variable genes were selected. Cells were filtered based on nFeature_RNA, retaining those with more than 100 and fewer than 5000 features. The “ScaleData” function was employed to regress out the effects of UMI counts and the percentage of mitochondrial-derived UMIs. Subsequently, major cell clusters were identified using the “FindClusters” function in Seurat. Unbiased cell type identification was achieved through Uniform Manifold Approximation and Projection (UMAP). To characterize specific marker genes for individual cell clusters, the “FindMarkers” function in Seurat was utilized to compare the gene expression profiles of cells within a given cluster against those of all other cells in the dataset. This function employs the Wilcoxon rank-sum test to identify differentially expressed genes (DEGs) between the two groups. The resulting p-values were then adjusted for multiple testing using the Bonferroni correction, accounting for the total number of genes analyzed. Marker genes were defined as those with an adjusted p-value below 0.05 and exhibiting at least a two-fold higher average expression level in the cluster of interest compared to all other clusters. To validate the annotation of each cell cluster, we relied on the expression of canonical marker genes. Specifically, T/NK cells were identified by the expression of CD3D, CD3E, and NKG7; memory B cells were characterized by CD79A and MS4A1. Myeloid cells were identified through the expression of CD68, CD163, CD14, LYZ, CD74, CLEC9A, and CD1C. Stromal cells were distinguished by high expression of COL1A1, ACTA2, and TAGLN. On the other hand, tumor cells were identified by the expression of CCR7, LEF1, and TCP7. This comprehensive analysis, leveraging statistical tests and the expression of established marker genes, enabled accurate annotation and characterization of each cell cluster. The expression levels and distribution of genes were visualized using FeaturePlot and ggplot2.

### Cell lines and cell culture

The cell lines used in this study were authenticated and provided by Dr. Zeng from the Cancer Center of Sun Yat-sen University. The human nasopharyngeal carcinoma (NPC) cell line, HONE1, was cultured in Roswell Park Memorial Institute (RPMI)-1640 medium (Invitrogen, Carlsbad, CA, USA), supplemented with 10% fetal bovine serum (FBS; Yingjie Biotechnology).

### Cell proliferation, migration, invasion, and colony formation assays

Cell Proliferation Assay: Cell proliferation was assessed using the Cell Counting Kit-8 (CCK-8) (Dojindo Laboratories, Kumamoto, Japan). A total of 1 × 10^3^ cells per well were seeded into 96-well plates, and cell viability was measured every 24 h for a period of 5 days, following the manufacturer’s instructions.

Transwell Migration and Invasion Assays: For the migration and invasion assays, 5 × 10^4^ cells (for migration) or 1 × 10^4^ cells (for invasion) were seeded in the upper chamber of Transwell inserts (BD Biosciences, San Jose, CA, USA) in serum-free medium. For the invasion assay, the chamber was pre-coated with Matrigel (BD Biosciences) to mimic the extracellular matrix. The lower chamber was filled with medium containing 10% FBS as a chemoattractant. After 24 h of incubation, cells that had migrated or invaded through the membrane were fixed, stained with hematoxylin, and visualized under an inverted microscope.

Colony Formation Assay: To assess clonogenic survival, single-cell suspensions (200–10,000 cells per well) were seeded into 6-well plates and treated with QZD (10 mg/mL). After 10–14 days, once colonies had formed, the wells were washed with PBS, fixed with methanol, and stained with crystal violet. Colonies consisting of more than 50 cells were counted to evaluate the treatment effect.

### Western blot analysis

Total protein was extracted from HONE1 cells using RIPA buffer (with protease and phosphatase inhibitors). After lysis, proteins were separated by SDS-PAGE and transferred to PVDF membranes. The membranes were blocked with 5% non-fat milk in TBST, then incubated overnight with primary antibodies (e.g., anti-AKT1, 1:1000, Cell Signaling Technology) at 4°C. After washing, membranes were incubated with HRP-conjugated secondary antibodies (1:2000, Thermo Fisher Scientific) for 2 h at room temperature. Proteins were detected using chemiluminescence (ECL, GE Healthcare) and imaged with the FlourChen M system. Band intensity was quantified using ImageJ software (version 1.53c).

### Quantitative real-time PCR (qPCR) analysis

Total RNA was extracted from cells using TRIzol reagent (Invitrogen) following the manufacturer’s protocol. Complementary DNA (cDNA) was synthesized using the HiScript III RT SuperMix (Vazyme, Nanjing, China) according to the supplier’s instructions. Quantitative real-time PCR (qPCR) was performed using the CFX96 Touch Real-Time PCR System (Bio-Rad, Hercules, CA, USA) with SYBR Green qPCR SuperMix-UDG (Invitrogen). Each sample was analyzed in triplicate, and the threshold cycle (Cq) values were recorded. Gene expression levels were normalized to glyceraldehyde-3-phosphate dehydrogenase (GAPDH) as an internal control. The primer sequences used for qPCR analysis are listed in Table 1.

**TABLE 1:** Primer sequences information.

Primer	Sequence (5′to3′)
IL6	AGA​CAG​CCA​CTC​ACC​TCT​TCA​G
SRC	CTG​CTT​TGG​CGA​GGT​GTG​GAT​G
ESR1	GCT​TAC​TGA​CCA​ACC​TGG​CAG​A
MTOR	AGC​ATC​GGA​TGC​TTA​GGA​GTG​G
AKT1	TGG​ACT​ACC​TGC​ACT​CGG​AGA​A
HIF1A	TAT​GAG​CCA​GAA​GAA​CTT​TTA​GGC

### Molecular docking analysis

The 2D structures of the core active components were downloaded from the PubChem database (https://pubchem.ncbi.nlm.nih.gov/) and converted into 3D structures using ChemBioOffice Ultra 13.0.2-Chem3D. Energy minimization was performed to optimize the structures. The 3D structures of the core target proteins were retrieved from the Protein Data Bank (PDB) (https://www.rcsb.org/) and preprocessed using PyMOL 2.5.2 to remove water molecules and irrelevant residues. The docking grid box was defined using AutoDockTools-1.5.7 to encompass the entire protein structure. Semi-flexible molecular docking was performed using AutoDock Vina (version 1.1.2), and the binding affinity (expressed as absolute binding energy) was calculated. Higher absolute binding energy values indicated stronger receptor-ligand interactions. The docking results were visualized and analyzed using PyMOL and Discovery Studio (version 4.5).

### Statistical analysis

All statistical analyses, including Wilcoxon rank-sum test, and ANOVA, were carried out in R (version 4.2.0). Statistical significance was defined as p < 0.05. Data visualization, including bar charts, point charts, line graphs, and other figures, was generated using the ggplot2 package (version 3.4.2), while heatmaps were constructed with the Heatmap package (version 2.14.0).

## Results

### UHPLC-OE-MS analysis and network construction

The QZD sample was obtained from the Traditional Chinese Medicine Pharmacy of Sun Yat-sen University Cancer Prevention and Treatment Center. The QZD was prepared by decocting 1,600 mL of water until it reached a rolling boil, then simmering over low heat until the volume was reduced to 200 mL, with a total decoction time exceeding 2 h. A second extraction was performed by adding 800 mL of water to the remaining dregs, boiling again, and simmering over low heat until the volume was reduced to 100 mL. The two extracts were combined for use.

**FIGURE 1:**
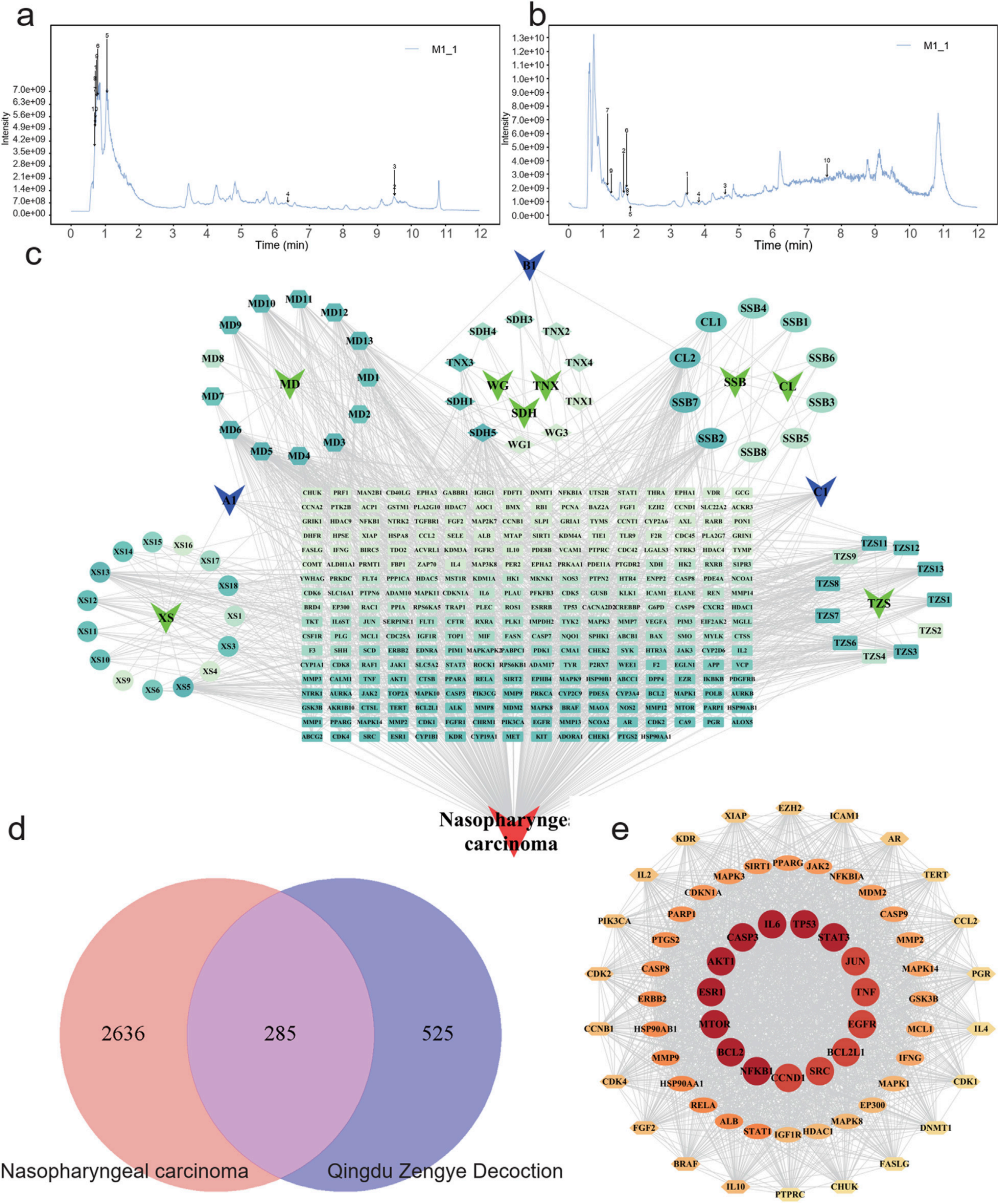
Characterization and network establishment of Qingdu Zengye Decoction. Total ion flow plots of QZD under different ionization modes. **(a)** Positive ion mode. **(b)** Negative ion mode. **(c)** QZD ingredient-target-disease network. Round nodes represent disease targets, Chinese medicine, and Chinese medicine active ingredients, and black edges represent the association between ingredients, targets, drugs, and diseases. **(d)** Venn diagram of overlapping target genes between QZD ingredients and NPC. **(e)** STRING database was used to analyze the protein-protein interaction (PPI) network of QZD for treating inflammation. The size and color of the nodes in the diagram are related to the degree in the network.

The primary components of QZD were analyzed using UHPLC-OE-MS (Supplementary Table S2). Representative ion chromatograms are shown in Figures 1a,b.

To explore the pharmacological mechanisms of QZD, we integrated target information from both its active compounds and NPC-related pathogenic genes. The overlapping targets were mapped and visualized in Cytoscape 3.10.0 to construct a “compound-target-disease” network, as shown in Figure 1c. This network consists of 356 nodes and 1408 edges, illustrating the complex multi-component, multi-target interactions between the decoction and the disease. Notably, a single active compound may interact with multiple targets, while different compounds may share the same target. Among them, the five compounds with the highest degree values (degree ranking, indicating their extensive target interactions) were MD5, SDH5, XS5, TZS3, SSB2 and MD6 (Figure 1c; Table 2; Supplementary Table S3).

**TABLE 2:** The degree value of the key Compound.

Number	Compound	Degree
1	MD5	47
2	SDH5	47
3	XS5	46
4	TZS3	46
5	MD6	46
6	SSB2	45
7	TZS11	44
8	MD4	44
9	MD3	43
10	SSB7	41

### PPI network and core targets

To identify core therapeutic targets of QZD for NPC treatment, we integrated data from multiple databases, and identified 285 overlapping anti-NPC targets. A protein-protein interaction (PPI) network was then constructed by importing these 285 targets into the STRING database. The resulting network contained 284 nodes and 6901 edges, with the top 14 nodes ranked by degree centrality listed in Table 3. These nodes were densely interconnected, highlighting their potential role as key regulatory hubs in NPC treatment (Figures 1d,e; Table 3).

**TABLE 3:** The degree value of the key target.

Number	Target	Degree
1	AKT1	64
2	ESR1	64
3	CASP3	64
4	IL6	64
5	BCL2	64
6	TP53	64
7	STAT3	64
8	NFKB1	64
9	MTOR	64
10	TNF	63
11	EGFR	63
12	BCL2L1	63
13	SRC	63
14	JUN	63

### Exploring the potential pathogenic mechanisms of nasopharyngeal carcinoma

**FIGURE 2:**
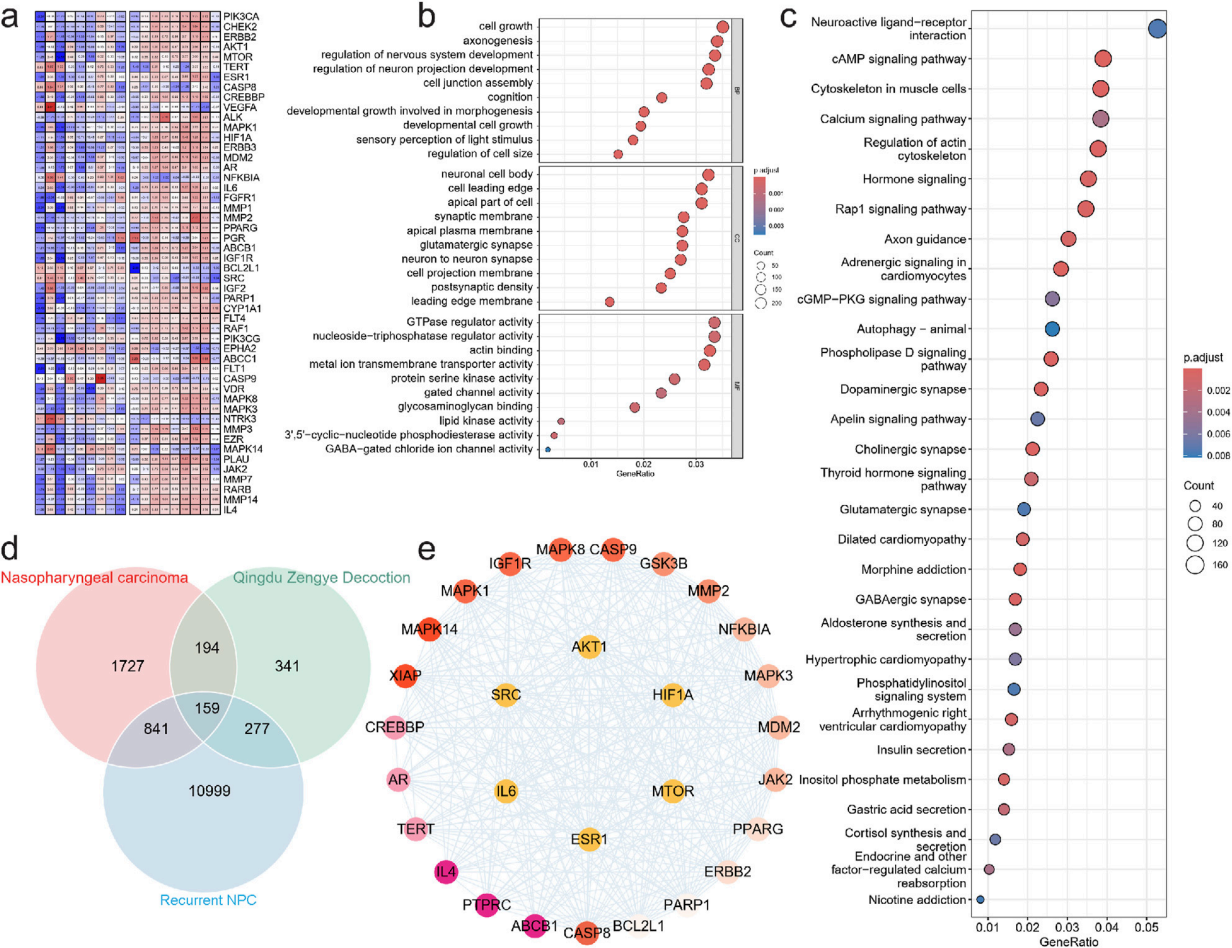
Experimental verification of key targets. **(a)** Signal intensity heatmap of methylation sequencing in patients with nasopharyngeal carcinoma. GO **(b)** and KEGG **(c)** enrichment analysis was performed to examine the possible functions of differentially methylated genes in NPC sample. **(d)** venn diagram of intersection of Nasopharyngeal carcinoma target gene, QZDtarget gene and Relapse related gene. **(e)** Protein interaction network of key targets.

To investigate the underlying mechanisms of high-risk locally advanced NPC, we first performed differential methylation analysis on methylation sequencing data (Figure 2a). The results revealed that DMGs were significantly enriched in biological processes such as regulation of neuron projection development, regulation of nervous system development, and axonogenesis, which are known to be involved in neural development and cellular signaling pathways. These findings suggest that alterations in cellular structure and signal transduction in high-risk locally advanced tumors may contribute to increased invasiveness and disease progression.

Additionally, DMGs were highly enriched in cellular components related to synaptic membrane, apical part of the cell, cell leading edge, and neuronal cell body, indicating that modifications in these structural components may facilitate tumor cell migration and invasion. This is particularly relevant as enhanced cellular motility is a hallmark of tumor aggressiveness and progression.

At the molecular function level, DMGs were significantly associated with metal ion transmembrane transporter activity, actin binding, triphosphatase regulator activity, and GTPase regulator activity, highlighting the role of cytoskeletal dynamics and intracellular signaling in tumor cell behavior. Dysregulation of these activities may enhance tumor adaptation to the microenvironment and enable evasion of therapy-induced stress (Figure 2b).

Pathway enrichment analysis further confirmed the involvement of multiple signaling pathways, including Axon guidance, Rap1 signaling pathway, Hormone signaling, Regulation of actin cytoskeleton, Calcium signaling pathway, Cytoskeleton in muscle cells, cAMP signaling pathway, and Neuroactive ligand-receptor interaction (Figure 2c). These pathways are known to regulate cell adhesion, motility, and proliferation, all of which are crucial for tumor progression. Notably, the repeated enrichment of actin cytoskeleton regulation underscores its pivotal role in high-risk locally advanced NPC, potentially by enhancing the migration and invasiveness of residual tumor cells following treatment. We then identified key regulatory proteins by intersecting NPC- related genes, QZD treatment targets, and high-risk locally advanced NPC-associated genes, resulting in 159 key proteins. Protein-protein interaction (PPI) analysis was performed to extract core regulatory proteins, revealing that AKT1, MTOR, HIF1A, IL6, SRC, and ESR1 serve as central regulatory targets (Figures 2d,e). These core proteins are well-known mediators of tumor survival, proliferation, and metastasis, suggesting that their dysregulation may play a critical role in high-risk locally advanced NPC progression and resistance to treatment.

We then identified key regulatory proteins by intersecting NPC-related genes, QZD treatment targets, and high-risk locally advanced NPC-associated genes, resulting in 159 key proteins. Protein-protein interaction (PPI) analysis was performed to extract core regulatory proteins, revealing that AKT1, MTOR, HIF1A, IL6, SRC, and ESR1 serve as central regulatory targets. These core proteins are well-known mediators of tumor survival, proliferation, and metastasis, suggesting that their dysregulation may play a critical role in high-risk locally advanced NPC progression and resistance to treatment.

### Pathway enrichment and single-cell transcriptomic analysis on QZD’s mechanisms of action in NPC after GP chemotherapy

**FIGURE 3:**
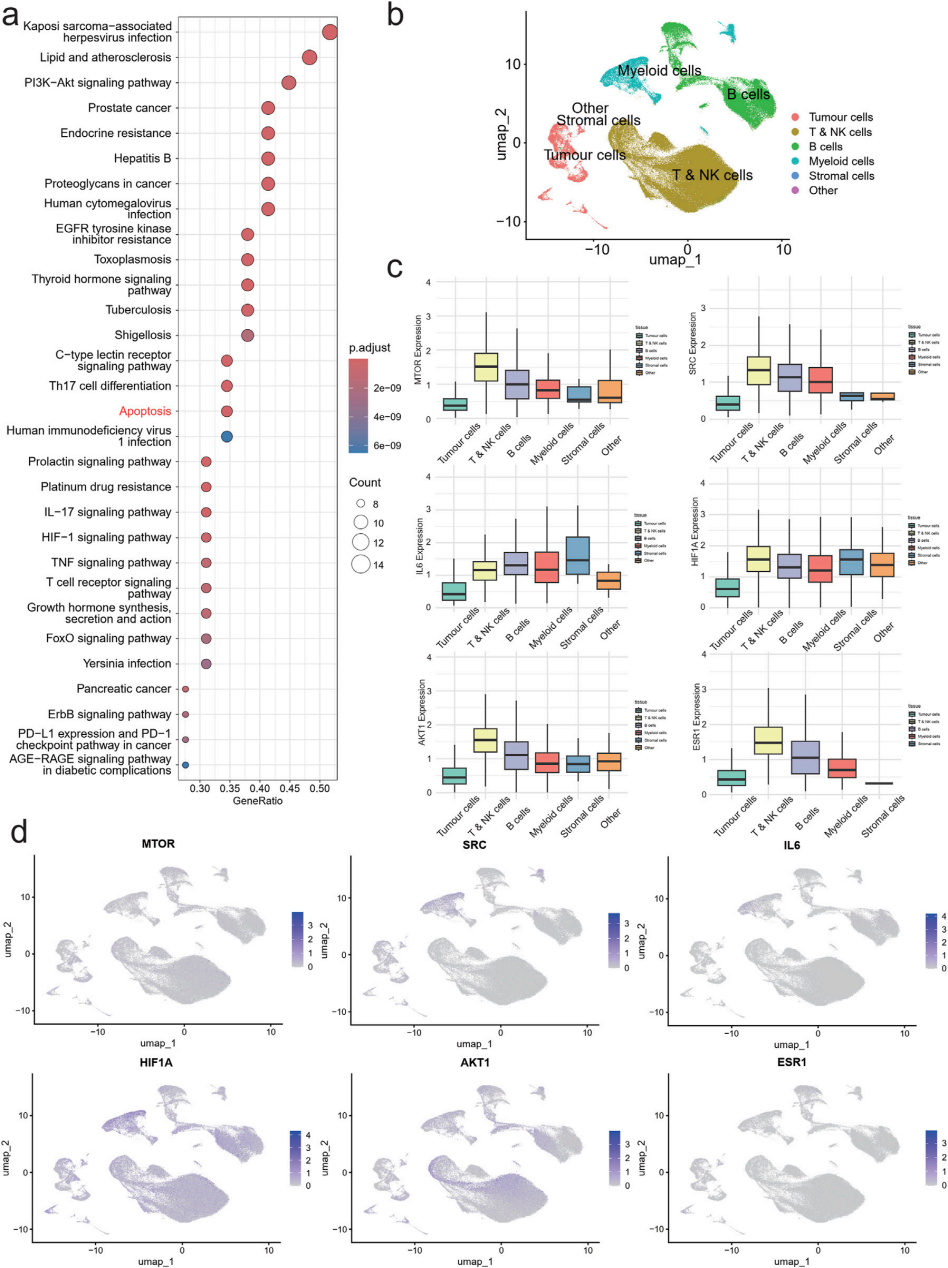
Single cell atlas of NPC. UMAP of 179,272 single cell transcriptomes (points), with similar cells positioned closer together. **(a)** KEGG enriched bubble plot. **(b)** UMAP plot of NPC colored by cell Type. **(c)** Boxplots of expression levels of AKT1, MTOR, HIF1A, IL6, SRC and ESR1 in each cell subpopulation. **(d)** UMAP plot of AKT1, MTOR, HIF1A, IL6, SRC and ESR1 expression in NPC cells.

Pathway enrichment analysis of the 159 key proteins identified from the intersection of NPC-related genes, QZD treatment targets, and high-risk locally advanced NPC-associated genes revealed that the primary intervention pathways of QZD in high-risk locally advanced NPC patients include Apoptosis, PI3K-Akt signaling pathway, Th17 cell differentiation, C-type lectin receptor signaling pathway, Thyroid hormone signaling pathway, EGFR tyrosine kinase inhibitor resistance, Proteoglycans in cancer, Endocrine resistance, Prostate cancer, and Lipid and atherosclerosis (Figure 3a).

These findings suggest that QZD may exert therapeutic effects in high-risk locally advanced NPC by modulating multiple oncogenic pathways, particularly through apoptosis regulation and PI3K-Akt signaling inhibition, which are crucial for tumor survival and drug resistance. Additionally, the impact of QZD on immune-related pathways, such as Th17 cell differentiation and C-type lectin receptor signaling, indicates a potential role in modulating the tumor microenvironment to counteract disease progression and immune evasion.

To investigate the tumor immune microenvironment (TIME) in high-risk locally advanced NPC following GP chemotherapy, we analyzed single-cell RNA sequencing (scRNA-seq) data from 30 NPC patient tumor samples obtained from our hospital. Using the well-established 10X Genomics Chromium-based scRNA-seq platform, we successfully captured the transcriptomes of 179,272 single cells. These cells were classified into six major populations: Tumor cells, T & NK cells, B cells, Myeloid cells, Stromal cells, and Other (Figure 3b).

Expression analysis of key regulatory genes (AKT1, MTOR, HIF1A, SRC, and ESR1) revealed their predominant localization within the T & NK cell population, while IL6 was mainly enriched in the Myeloid compartment (Figure 3c). UMAP distribution showed that AKT1 and ESR1 were primarily expressed in tumor cells, SRC and IL6 were concentrated in the Myeloid subpopulation, while MTOR and HIF1A exhibited a broader distribution across multiple cell types (Figure 3d).

These findings suggest that QZDmay influence high-risk locally advanced NPC by modulating key signaling molecules within specific immune and tumor cell populations. The localization of AKT1 and ESR1 in tumor cells, alongside SRC and IL6 in myeloid cells, highlights potential mechanisms through which the decoction impacts tumor-immune interactions, potentially altering immune surveillance and tumor progression in high-risk locally advanced NPC.

### QZD inhibits NPC progression by inducing cell apoptosis and targeting key signaling pathways

**FIGURE 4:**
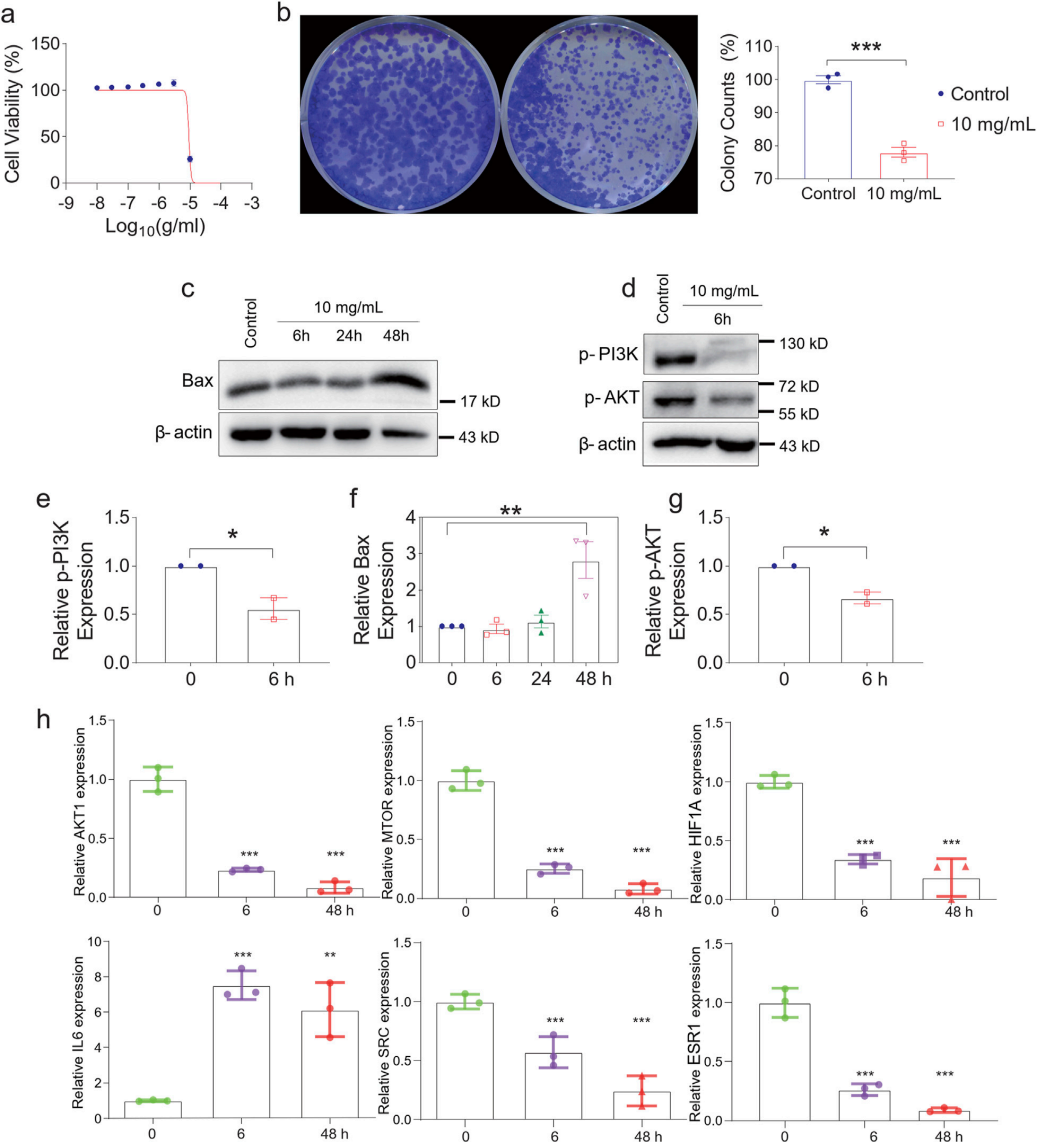
Experimental verification of key targets. **(a)** Cell Viability of HONE-1 at different concentrations. **(b)** QZDsignificantly reduced the growth rate of the indicated cells. **(C)** Western blotting analysis of Bax **(c)**, p-PI3K, p-AKT **(d)** and β-actin expression in HONE-1. mRNA expression levels of p-PI3K **(e)**, Bax **(f)**, p-AKT **(g)** in nasopharyngeal carcinoma tissue samples. **(h)** The levels of AKT1, MTOR, HIF1A, IL6, SRC and ESR1 expression with the time of Qingdu Zengye Decoction. *P < 0.05, **P < 0.01, ***P < 0.001.

Building on our previous pathway enrichment analysis, we identified that QZDprimarily targets apoptosis-related signaling pathways, suggesting its potential anti-tumor effects. To further validate its therapeutic role in NPC, particularly through apoptosis activation and modulation of AKT1, MTOR, HIF1A, SRC, and ESR1, we evaluated cell viability upon QZD treatment. Our results demonstrated a dose-dependent decrease in cell viability with increasing concentrations of QZD (Figure 4a). Additionally, colony formation assays revealed a significant reduction in colony counts, supporting the inhibitory effect of the decoction on NPC cell proliferation (Figure 4b).

To investigate the mechanisms of apoptosis, we examined the expression of Bax, a pro-apoptotic marker, over time. Bax levels initially decreased at 6h and 24h, likely due to drug effect, but rebounded at 48h, suggesting a sustained apoptotic response (Figure 4c). Concurrently, p-PI3K and p-AKT, two critical components of the PI3K-Akt signaling pathway, showed a significant reduction at 6h (Figure 4d), indicating early apoptosis induction.

Moreover, the expression levels of AKT1, MTOR, HIF1A, SRC, and ESR1 significantly declined over time, suggesting their involvement as key regulatory targets of QZD. Interestingly, IL6, known for its dual role as a pro-inflammatory cytokine and an anti-inflammatory myokine, exhibited a significant increase, suggesting a potential immune-modulatory effect of the decoction in the tumor microenvironment (Figures 4e–h).

**FIGURE 5:**
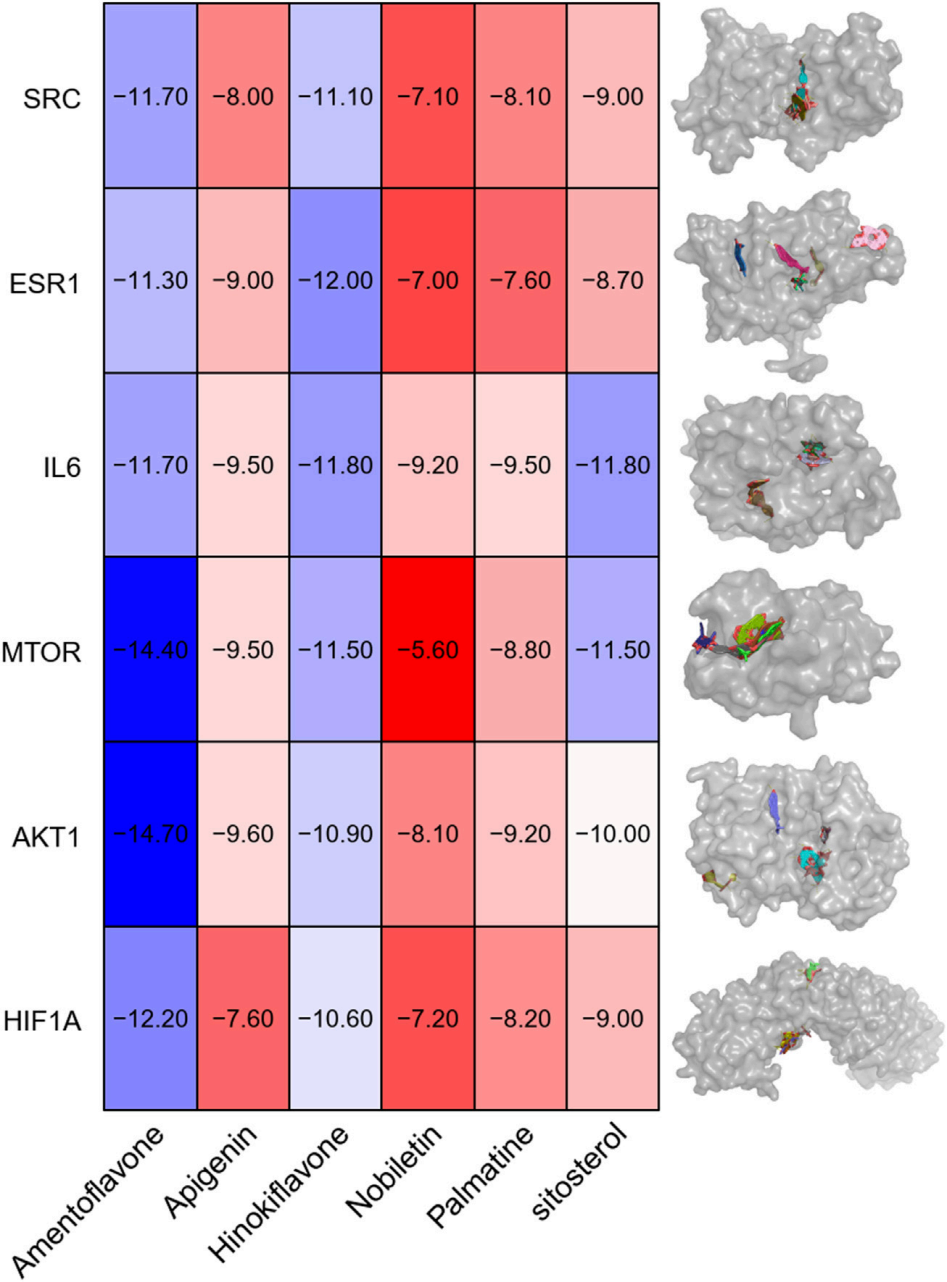
Visualization of molecular docking. Note: AKT1, MTOR, HIF1A, IL6(a), SRC and ESR1 and (1) Amentoflavone, (2) Apigenin, (3) Hinokiflavone, (4) Nobiletin, (5) Palmatine and (6) β-sitosterol.

The binding affinities of the previously obtained compounds with AKT1, MTOR, HIF1A, SRC, and ESR1 ranged from −14.7 to −5.6 (Figure 5). The docking results indicate that these small molecule compounds exhibit strong binding affinity with AKT1, MTOR, HIF1A, SRC, and ESR1.

**FIGURE 6:**
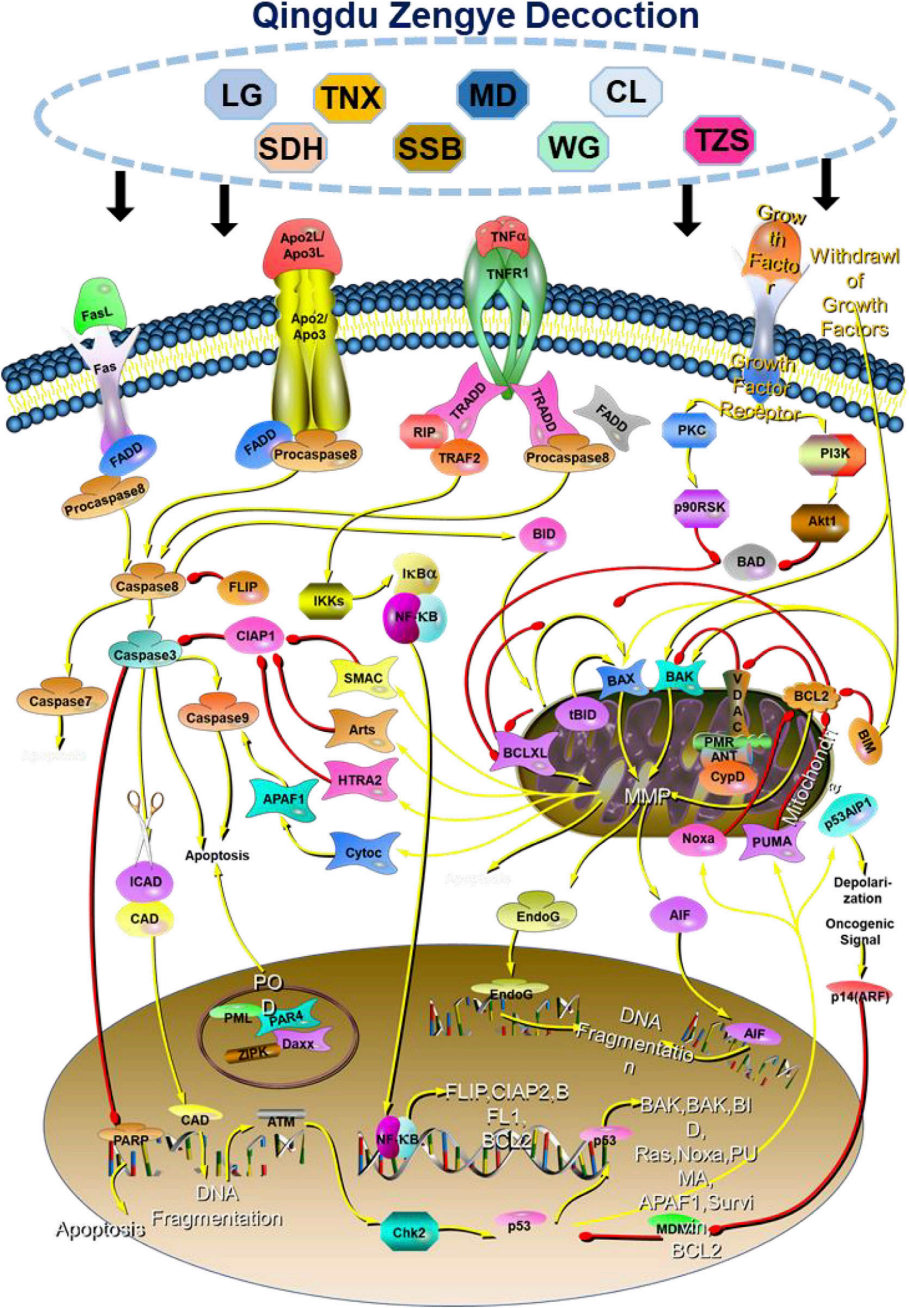
Mechanism of action of QZD for nasopharyngeal carcinoma.

Our findings demonstrate that QZD exerts its anti-tumor effects in high-risk locally advanced NPC primarily by inducing apoptosis and inhibiting the PI3K-Akt signaling pathway. QZD significantly reduces cell viability and colony formation in a dose-dependent manner while promoting apoptosis, as indicated by the Bax dynamics over time. The downregulation of p-PI3K and p-AKT confirms suppression of survival signaling, while the inhibition of key oncogenic regulators AKT1, MTOR, HIF1A, SRC, and ESR1 suggests broader effects on tumor metabolism, hypoxia adaptation, and growth factor receptor pathways. Additionally, the upregulation of IL6 may indicate an immune-mediated tumor suppression effect, warranting further investigation (Figure 6). These findings highlight QZD’s potential as a therapeutic intervention for high-risk locally advanced NPC.

## Discussion

Recurrence remains a significant challenge in NPC, with approximately 30% of high-risk locally advanced patients experiencing relapse or metastasis following standard chemoradiotherapy (Chen et al., 2019; Tang et al., 2022; Lee A. W. M. et al., 2019; Lin et al., 2024). While advances such as GP induction regimens and immune checkpoint inhibitors have improved outcomes, the limited tolerance to adjuvant chemotherapy and persistent recurrence risks highlight the urgent need for safer, more sustainable interventions (Dai et al., 2024; Chen Y. P. et al., 2021; Yip et al., 2023). In this context, QZD, a traditional Chinese medicine (TCM) formulation developed by our team at Sun Yat-sen University Cancer Center, has shown promising clinical utility in rescuing high-risk locally advanced NPC patients unresponsive to conventional therapies (Luo et al., 2024; Wang F. H. et al., 2021; Mai et al., 2021). A retrospective analysis of 91 post-chemoradiotherapy NPC cases treated with QZD demonstrated its favorable safety profile and its capacity to stabilize disease progression and extend survival (Huang et al., 2020). This research was supported by mechanistic studies revealing apoptosis induction and PI3K-Akt pathway inhibition. QZD’s multi-target efficacy, grounded in TCM principles of *qi-yin replenishment and phlegm-stasis resolution*, addresses key pathophysiological features of high-risk locally advanced NPC, such as residual phlegm-heat toxins and collateral obstruction (Xu et al., 2018; Chow et al., 2022). These findings align with growing clinical evidence supporting TCM’s role in mitigating recurrence risks through the modulation of oncogenic signaling and immune microenvironment dynamics. This work exemplifies how the integration of traditional herbal wisdom with modern oncology can bridge critical gaps in managing high-risk NPC.

Our results provide robust experimental validation for the clinical efficacy of QZD in combating high-risk locally advanced NPC. The dose-dependent suppression of NPC cell viability and significant reduction in colony formation underscore QZD’s potent anti-proliferative effects, which are crucial for halting disease progression in relapse-prone patients. Notably, the dynamic modulation of Bax—a transient decline at 6–24 h followed by a rebound at 48 h suggests a biphasic apoptotic mechanism. Early cellular adaptation to therapeutic stress is followed by sustained activation of intrinsic apoptosis, ultimately tipping the balance toward tumor cell elimination. This observation is consistent with clinical findings where QZD-treated patients exhibit delayed yet durable therapeutic responses, potentially overcoming the apoptosis resistance characteristic of high-risk locally advanced NPC.

Central to QZD’s efficacy is its dual blockade of the PI3K-Akt survival axis. The rapid downregulation of p-PI3K and p-AKT at 6 h disrupts a key pathway driving high-risk NPC progression (Wang S. et al., 2021; Zhao et al., 2016), while the progressive inhibition of downstream effectors (AKT1, MTOR, HIF1A, SRC, ESR1) amplifies this anti-tumor effect by interfering with metabolic adaptation, hypoxia signaling, and growth factor receptor cascades (Cai et al., 2019; Liu et al., 2024c; Li H. L. et al., 2022). These multi-layered disruptions are consistent with QZD’s TCM-based design, which targets the “phlegm-stasis-heat toxin” pathogenesis through compounds such as Pseudostellaria heterophylla (qi-tonifying) and Selaginella doederleinii (heat-clearing). Crucially, the observed IL6 upregulation—a paradoxical yet consistent finding—suggests that QZD may modulate TME. While IL6 is traditionally considered pro-tumorigenic, its elevation in this study may reflect immune activation, possibly enhancing myeloid cell-mediated tumor surveillance or counteracting T-cell exhaustion, as indicated by scRNA-seq localization of IL6 in myeloid compartments.

The clinical implications of these findings are profound. For high-risk locally advanced NPC patients—a group with limited therapeutic options and poor prognosis—QZD offers a multi-target strategy that not only induces apoptosis directly but also dismantles the molecular infrastructure supporting tumor survival and immune evasion (Ghi and Paccagnella, 2019; Liu et al., 2021; Wang et al., 2023). By suppressing HIF1A and MTOR, QZD may impair hypoxia-driven angiogenesis and metabolic reprogramming, both of which are hallmarks of post-chemoradiotherapy recurrence. Additionally, its inhibition of SRC and ESR1 could mitigate epithelial-mesenchymal transition (EMT) and hormone receptor-mediated growth, both of which are often exploited in metastatic NPC (Lasagna et al., 2020; Loo et al., 2022). Importantly, the safety profile of QZD, as demonstrated in our previous study, supports its viability as an adjunct to modern therapies, particularly for individuals intolerant to conventional adjuvant chemotherapy (Yan et al., 2013).

These findings also align with broader trends in integrative oncology. While Western medicine typically emphasizes pathway-specific inhibitors, QZD exemplifies how TCM’s “multi-component, multi-target” approach can address the complexity of high-risk NPC. The strong binding affinities between QZD compounds and core targets validate its design rationale, bridging herbal pharmacology with molecular precision. Future studies should focus on isolating active constituents (e.g., MD5, SDH5) and optimizing combinatorial regimens with immunotherapies, leveraging QZD’s IL6-mediated immunomodulatory potential. For now, QZD stands as a testament to the translational power of harmonizing ancient herbal wisdom with modern oncobiology—a beacon of hope for NPC patients navigating the perilous waters of disease progression.

While this study provides comprehensive insights into the therapeutic mechanisms of QZD, several limitations must be acknowledged. First, the absence of *in vivo* animal models prevents direct validation of QZD’s efficacy within a systemic TME. However, our integration of clinical outcomes (91 cases), single-cell transcriptomics, methylation profiling, and *in vitro* functional assays offers robust, human-relevant evidence that aligns with observed clinical benefits. Notably, the direct use of patient-derived samples and clinical data enhances translational relevance, as animal models often fail to replicate the complexity of human high-risk locally advanced NPC, particularly in the context of TCM interventions. Second, although our omics approach identified key targets (e.g., AKT1, IL6) and pathways, further pharmacological studies are needed to resolve QZD’s pharmacokinetic properties and bioactive compound specificity. Future research could employ patient-derived xenograft (PDX) models or organoid systems to bridge this gap while preserving human TME fidelity. Despite these limitations, our findings lay a critical foundation for advancing QZD as a clinically viable option for high-risk locally advanced NPC, supported by its safety profile, multi-target efficacy, and mechanistic alignment with TCM principles.

## Conclusion

This study highlights the potential of QZD as a promising multi-target therapeutic strategy for high-risk locally advanced NPC. By integrating TCM principles with modern molecular insights, the research showcases how QZD can target complex tumorigenic networks. Through network pharmacology, single-cell transcriptomics, and functional assays, we demonstrate that QZD suppresses NPC progression via apoptosis induction, PI3K-Akt pathway inhibition, and modulation of the tumor immune microenvironment.

## Data Availability

The scRNA-seq sequencing data generated by this study were deposited at the CNGB Sequence Archive (https://db.cngb.org/cnsa/) under accession nos. CNP0001341 for the scRNA-seq raw data and CNP0001503 for the scRNA-seq processed data. Raw FASTQ data are publicly available as of the date of publication. The names of the repository/repositories and accession number(s) can be found in the article/Supplementary Material. All raw data are also available from the corresponding authors upon reasonable request.

## Glossary

NPC: Nasopharyngeal carcinoma
EBV: Epstein-Barr virus
CCRT: Concurrent chemoradiotherapy
PF: Cisplatin + fluorouracil
GP: Gemcitabine + cisplatin
PD-1: Programmed cell death protein 1
TCM: Traditional Chinese Medicine
QZD: Qingdu Zengye Decoction
GO: Gene Ontology
KEGG: Kyoto Encyclopedia of Genes and Genomes
DMG: Differentially methylated genes
PPI: Protein-protein interaction
TSS: Transcription start site
UTR: Untranslated region
LC-MS: Liquid chromatography-mass spectrometry
CCK-8: Cell Counting Kit-8
PBS: Phosphate-buffered saline
RPMI: Roswell Park Memorial Institute
FBS: Fetal bovine serum
SDS-PAGE: Sodium dodecyl sulfate polyacrylamide gel electrophoresis
RT: Reverse transcription
PCR: Polymerase chain reaction
qPCR: Quantitative polymerase chain reaction
UMIs: Unique molecular identifiers
UMAP: Uniform manifold approximation and projection
IL6: Interleukin 6
AKT: Protein kinase B
MTOR: Mechanistic target of rapamycin
HIF1A: Hypoxia-inducible factor 1-alpha
SRC: Proto-oncogene c-Src
ESR1: Estrogen receptor 1
RCF: Relative centrifugal force
RPM: Revolutions per minute
PDX: Patient-derived xenograft
MS/MS: Tandem mass spectrometry
