# Supplementary material for: Uncovering the molecular mechanisms of Qingdu Zengye Decoction in the treatment of nasopharyngeal carcinoma: an integrative investigation
Source: Front Pharmacol. 2025 Jul 17;16:1648294. doi: 10.3389/fphar.2025.1648294 (PMC12310586; doi:10.3389/fphar.2025.1648294)
Supplement: Supplementary file 1 [file Table1.docx]

**Supplementary Table1 Clinical characteristics of Patients with NPC**

| **Variables** | | **Total** | **non-recurrent group** | **recurrent**  **group** |
| --- | --- | --- | --- | --- |
| Sex | male | 10 | 5 | 5 |
|  | female | 8 | 4 | 4 |
| Age | range | 31-69 | 31-66 | 35-69 |
| Family History | yes | 7 | 3 | 4 |
|  | no | 11 | 6 | 5 |
| T stage | 1 | 1 | 1 | 0 |
|  | 2 | 5 | 3 | 2 |
|  | 3 | 12 | 5 | 7 |
| N stage | 0 | 1 | 0 | 1 |
|  | 1 | 12 | 8 | 4 |
|  | 2 | 4 | 1 | 3 |
|  | 3 | 1 | 0 | 1 |

**Supplementary Table 2 The main ingredients of QZD obtained by UHPLC-OE-MS**

| **neg_tic_label** | | | | |
| --- | --- | --- | --- | --- |
| **ID** | **MS2.name** | **Formula** | **mz** | **rt** |
| 1 | DL-Threonine | C4H9NO3 | 118.051 | 42.7 |
| 2 | Oleic acid | C18H34O2 | 281.2489 | 570.3 |
| 3 | trans-Vaccenic acid | C18H34O2 | 281.2489 | 570.3 |
| 4 | 1-Naphthol | C10H8O | 143.0504 | 382.3 |
| 5 | Hydroxyacetone | C3H6O2 | 73.0296 | 63.2 |
| 6 | Glyceraldehyde | C3H6O3 | 89.0245 | 47 |
| 7 | Sarcosine | C3H7NO2 | 88.0405 | 42 |
| 8 | Alanine | C3H7NO2 | 88.0405 | 42 |
| 9 | Trehalose | C12H22O11 | 341.1089 | 45.1 |
| 10 | L-Arginine | C6H14N4O2 | 173.1045 | 41.2 |
| **pos_tic_label** | | | | |
| **ID** | **MS2.name** | **Formula** | **mz** | **rt** |
| 1 | DL-Tryptophan | C11H12N2O2 | 205.0971 | 208.2 |
| 2 | Inosine | C10H12N4O5 | 269.0878 | 96.5 |
| 3 | Isoorientin | C21H20O11 | 449.1075 | 275.2 |
| 4 | gamma-Glutamylphenylalanine | C14H18N2O5 | 295.1289 | 228.7 |
| 5 | Phloroglucinol | C6H6O3 | 127.0388 | 108.1 |
| 6 | Guanosine | C10H13N5O5 | 284.0986 | 101.6 |
| 7 | Leucine | C6H13NO2 | 132.1017 | 67.9 |
| 8 | Kynurenine | C10H12N2O3 | 209.0918 | 103 |
| 9 | Adenine | C5H5N5 | 136.0616 | 74.2 |
| 10 | Sciadopitysin | C33H24O10 | 581.1429 | 454.8 |

**Supplementary Table 3 The main ingredients of QZD**

| Code | Ingredient | Medicinal materials |
| --- | --- | --- |
| A1 | beta-sitosterol | Rhizoma Arisaematis、Pseudostellaria heterophylla、Scrophularia ningpoensis |
| B1 | sitosterol | Rhizoma Arisaematis、Selaginella doederleinii Hieron、Scrophularia ningpoensis |
| C1 | diosgenin | Rhizoma Paridis、Ophiopogon japonicus |
| SDH1 | coniferin | Rehmannia glutinosa |
| SDH2 | adenine | Rehmannia glutinosa |
| SDH3 | 3-indolecarboxylic acid | Rehmannia glutinosa |
| SDH4 | rehmapicrogenin | Rehmannia glutinosa |
| SDH5 | diincarvilone A | Rehmannia glutinosa |
| TNX1 | [(2R)-2-[[[(2R)-2-(benzoylamino)-3-phenylpropanoyl]amino]methyl]-3-phenylpropyl] acetate | Rhizoma Arisaematis |
| TNX2 | 24-epicampesterol | Rhizoma Arisaematis |
| TNX3 | Stigmasterol | Rhizoma Arisaematis |
| TNX4 | CLR | Rhizoma Arisaematis |
| SSB1 | Yangambin | Selaginella doederleinii Hieron |
| SSB2 | palmatine | Selaginella doederleinii Hieron |
| SSB3 | berberine | Selaginella doederleinii Hieron |
| SSB4 | nobiletin | Selaginella doederleinii Hieron |
| SSB5 | synephrine | Selaginella doederleinii Hieron |
| SSB6 | hordenine | Selaginella doederleinii Hieron |
| SSB7 | anisodamine | Selaginella doederleinii Hieron |
| SSB8 | hyoscyamine | Selaginella doederleinii Hieron |
| WG1 | histamine | Centipede |
| WG2 | leucine | Centipede |
| WG3 | tyrosin | Centipede |
| CL1 | flavone | Rhizoma Paridis |
| CL2 | Pennogenin | Rhizoma Paridis |
| MD1 | ophiopogonone b | Ophiopogon japonicus |
| MD2 | isoophiopogonone a | Ophiopogon japonicus |
| MD3 | ophiopogonanone a | Ophiopogon japonicus |
| MD4 | orchinol | Ophiopogon japonicus |
| MD5 | n-trans-feruloyltyramine | Ophiopogon japonicus |
| MD6 | ophiopogonanone b | Ophiopogon japonicus |
| MD7 | 5,7-dihydroxy-6,8-dime thyl-3-(4'-hydroxy-3'-methoxybenzyl)chroman-4-one | Ophiopogon japonicus |
| MD8 | ophiopogonone c | Ophiopogon japonicus |
| MD9 | ophiopogonanone c | Ophiopogon japonicus |
| MD10 | ophiopogonanone e | Ophiopogon japonicus |
| MD11 | ophiopogonanone d | Ophiopogon japonicus |
| MD12 | ophiopogonanone f | Ophiopogon japonicus |
| MD13 | ruscogenin | Ophiopogon japonicus |
| TZS1 | acacetin | Pseudostellaria heterophylla |
| TZS2 | Linarin | Pseudostellaria heterophylla |
| TZS3 | luteolin | Pseudostellaria heterophylla |
| TZS4 | Schottenol | Pseudostellaria heterophylla |
| TZS5 | 1-Monolinolein | Pseudostellaria heterophylla |
| TZS6 | isoeugenol | Pseudostellaria heterophylla |
| TZS7 | apoatropine | Pseudostellaria heterophylla |
| TZS8 | kaempferol | Pseudostellaria heterophylla |
| TZS9 | glutamic acid | Pseudostellaria heterophylla |
| TZS10 | multiflorine | Pseudostellaria heterophylla |
| TZS11 | multiradiatin | Pseudostellaria heterophylla |
| TZS12 | octadecanoic acid | Pseudostellaria heterophylla |
| TZS13 | dibutyl phthalate | Pseudostellaria heterophylla |
| TZS14 | guanine | Pseudostellaria heterophylla |
| XS1 | sugiol | Scrophularia ningpoensis |
| XS2 | 14-deoxy-12(R)-sulfoandrographolide | Scrophularia ningpoensis |
| XS3 | harpagoside_qt | Scrophularia ningpoensis |
| XS4 | cis-Zimtsaeure | Scrophularia ningpoensis |
| XS5 | liquiritigenin | Scrophularia ningpoensis |
| XS6 | 2-hydroxy-5-methoxyacetophenone | Scrophularia ningpoensis |
| XS7 | para-hydroxy-acetophenone | Scrophularia ningpoensis |
| XS8 | buergerinin b | Scrophularia ningpoensis |
| XS9 | buergerinin g | Scrophularia ningpoensis |
| XS10 | buergerinin f | Scrophularia ningpoensis |
| XS11 | ningposide b | Scrophularia ningpoensis |
| XS12 | ningposide d | Scrophularia ningpoensis |
| XS13 | linoleic acid | Scrophularia ningpoensis |
| XS14 | trans-palmitoleic acid | Scrophularia ningpoensis |
| XS15 | hexadecanoic acid | Scrophularia ningpoensis |
| XS16 | syringic acid | Scrophularia ningpoensis |
| XS17 | oleic acid | Scrophularia ningpoensis |
| XS18 | vanillic acid | Scrophularia ningpoensis |
